# Supplementary material for: Synthesis and Molecular Docking Study of Novel Pyrimidine Derivatives against COVID-19
Source: Molecules. 2023 Jan 11;28(2):739. doi: 10.3390/molecules28020739 (PMC9863666; doi:10.3390/molecules28020739)
Supplement: Supplementary file 1 [file molecules-28-00739-s001.zip › molecules-2076835-supplementary.pdf]

Supplementary File

Synthesis and Molecular Docking Study of Novel Pyrimidine Derivatives against COVID-19

Zahra M. Alamshany [1], Reham R. Khattab [2], Nasser A. Hassan [2], Ahmed A. El-Sayed [2,*], Mohamed A. Tantawy [3], Ahmed Mostafa [4] and Allam A. Hassan [5,*]

[1] Chemistry Department, Faculty of Science, King Abdulaziz University, Jeddah 21551, Saudi Arabia
[2] Photochemistry Department (Synthetic Unit), National Research Centre, Dokki, Giza 12622, Egypt
[3] Hormones Department, National Research Centre, Dokki, Giza 12622, Egypt
[4] Center of Scientific Excellence for Influenza Viruses, National Research Centre, Dokki, Giza 12622, Egypt
[5] Chemistry Department, Faculty of Science, Suez University, Suez 43221, Egypt
* Correspondence: ahmedcheme4@yahoo.com (A.A.E.-S.); allam.hassan@sci.suezuni.edu.eg (A.A.H.)

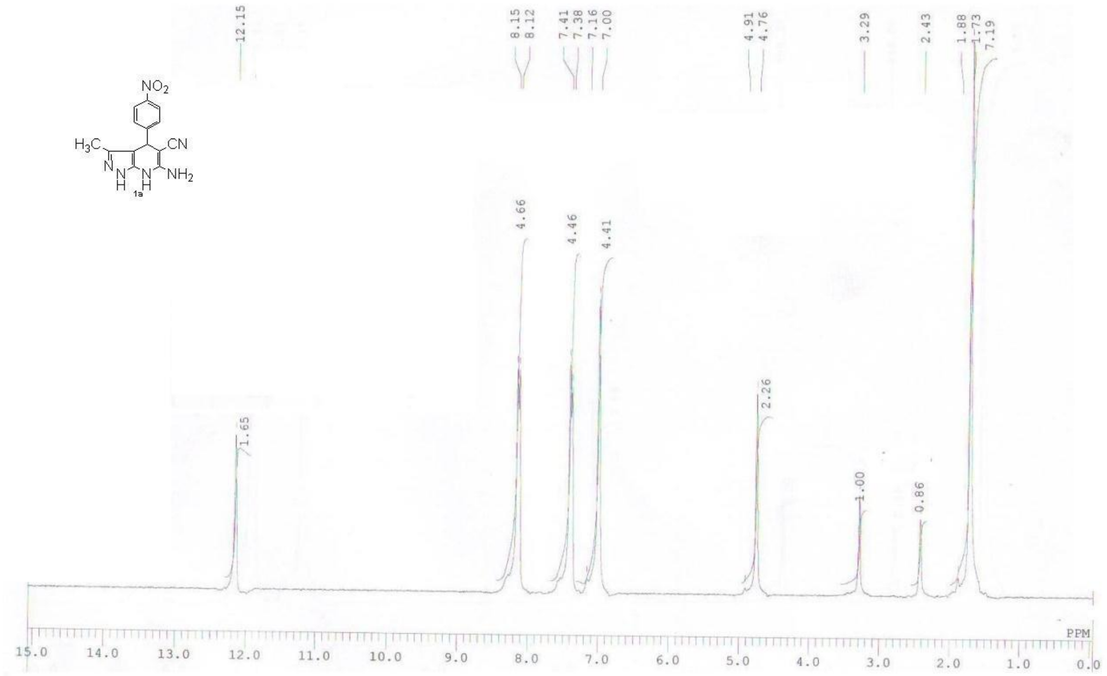

Chart S1: ^{1}H NMR of starting material.

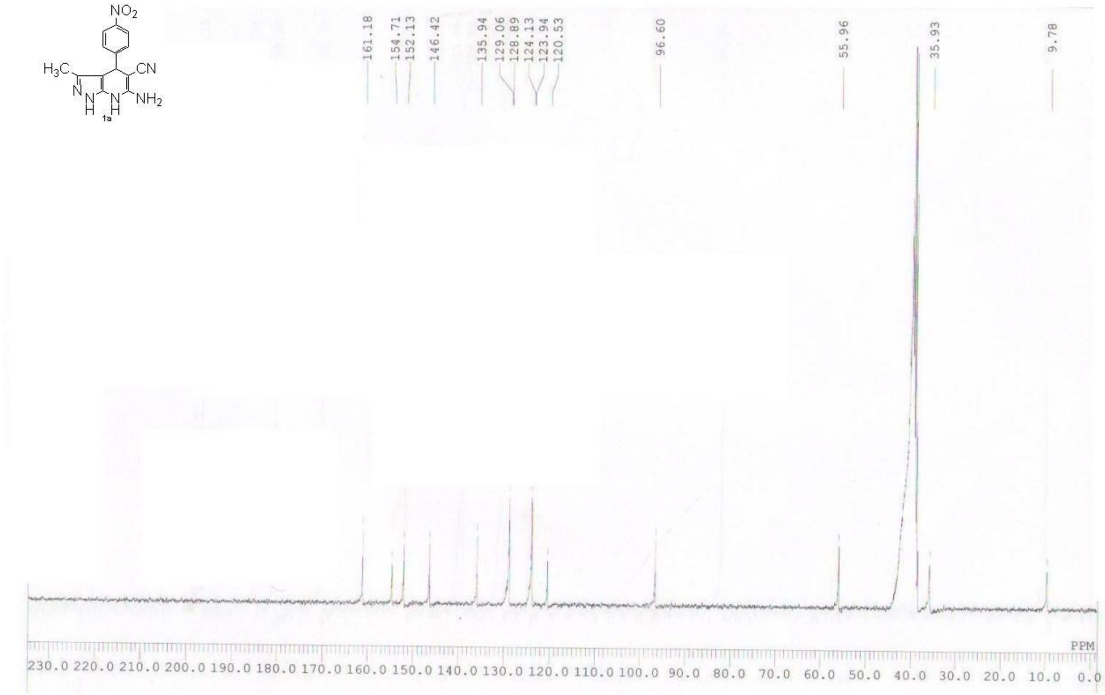

Chart S2: ^{1}H NMR of starting material.

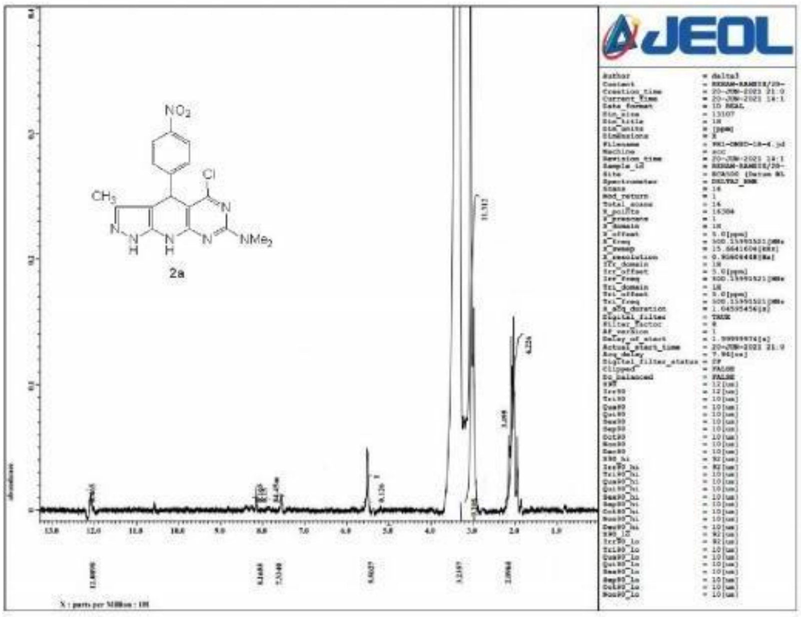

Chart S3: ^{1}H NMR of Compound **2a**.

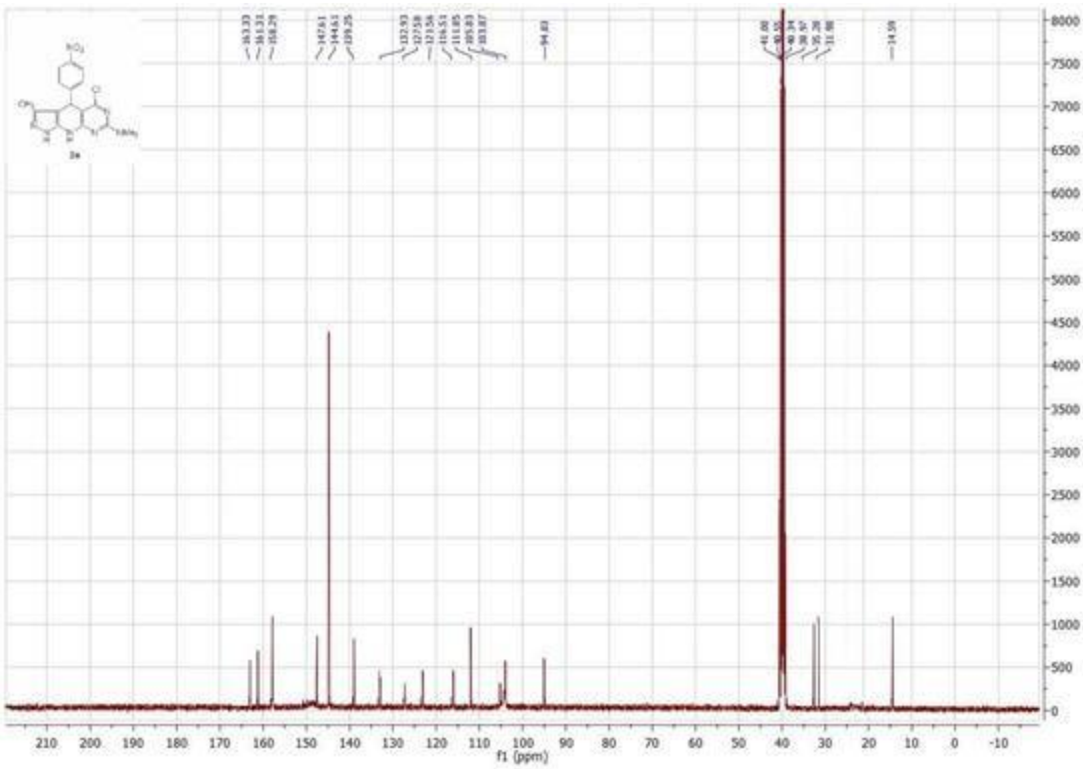

Chart S4: ^{13}C NMR of Compound **2a**.

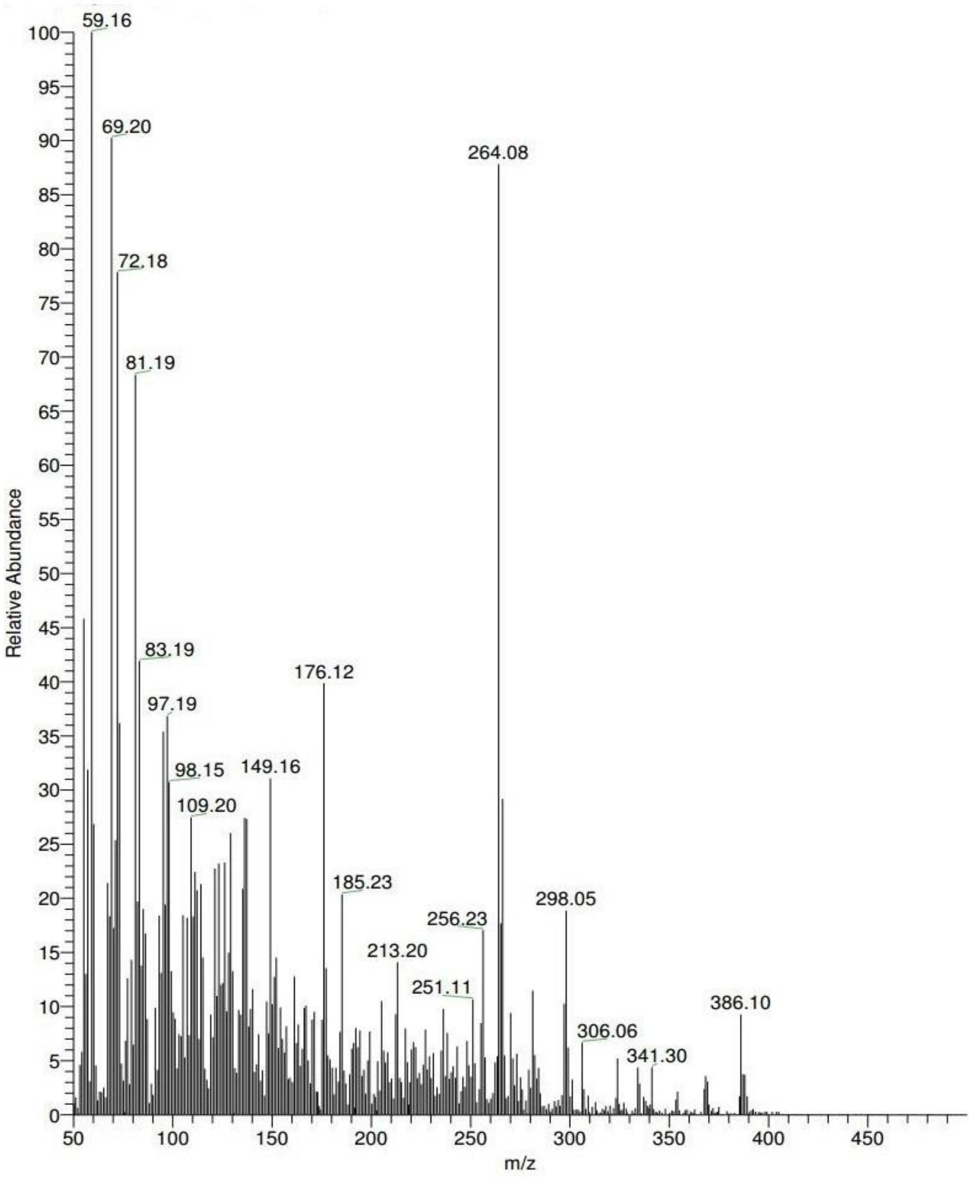

Chart S5: MS of Compound **2a.**

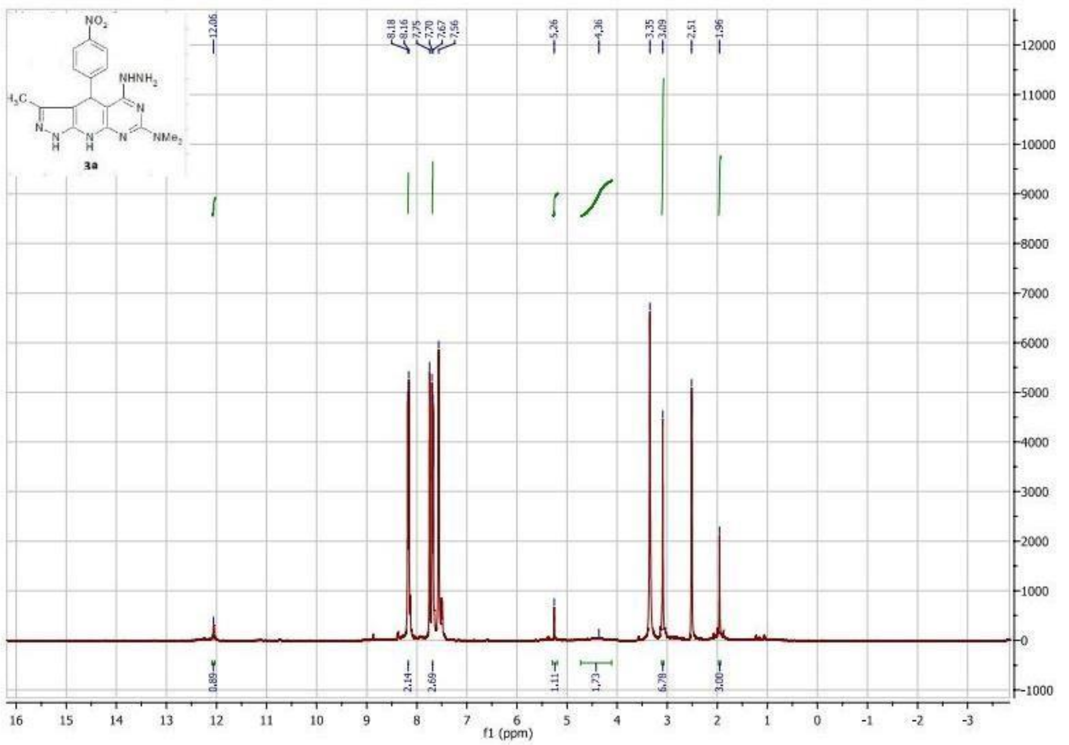

Chart S6: ^{1}H NMR of Compound **3a.**

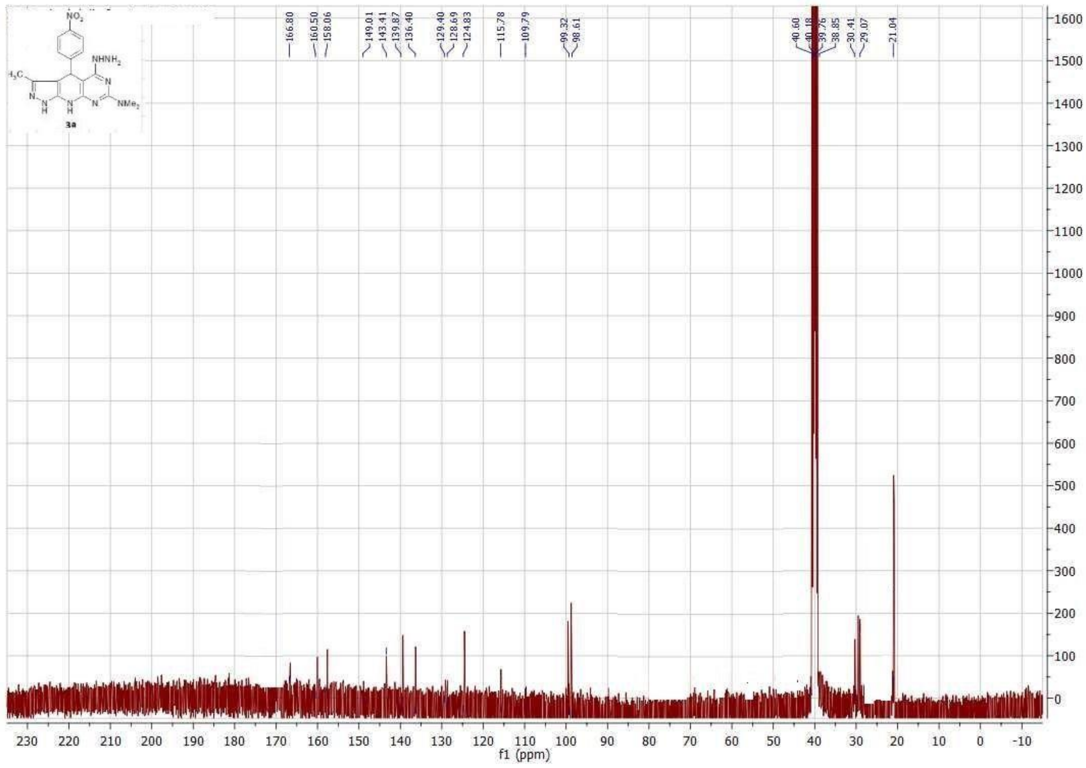

Chart S7: ^{13}C NMR of Compound **3a.**

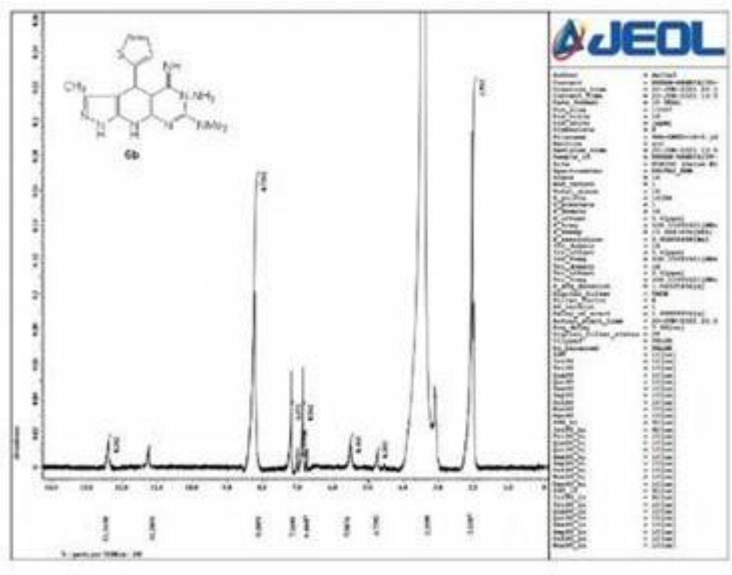

Chart S8: ^{1}H NMR of Compound **6b.**

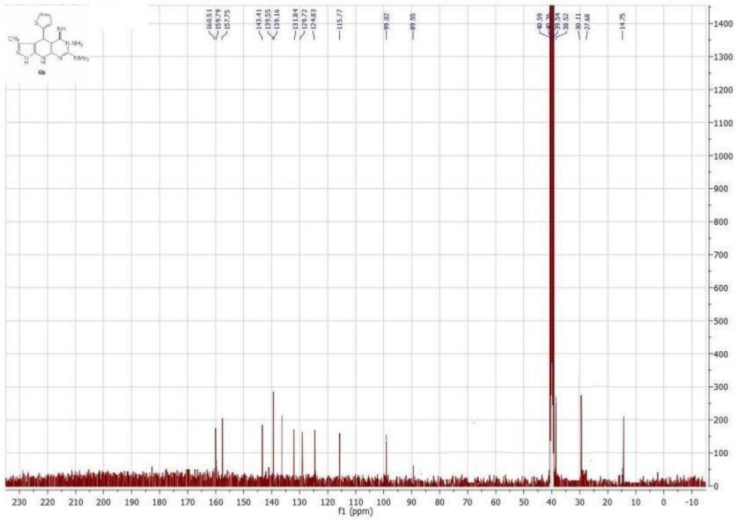

Chart S9: ^{13}C NMR of Compound **6b.**

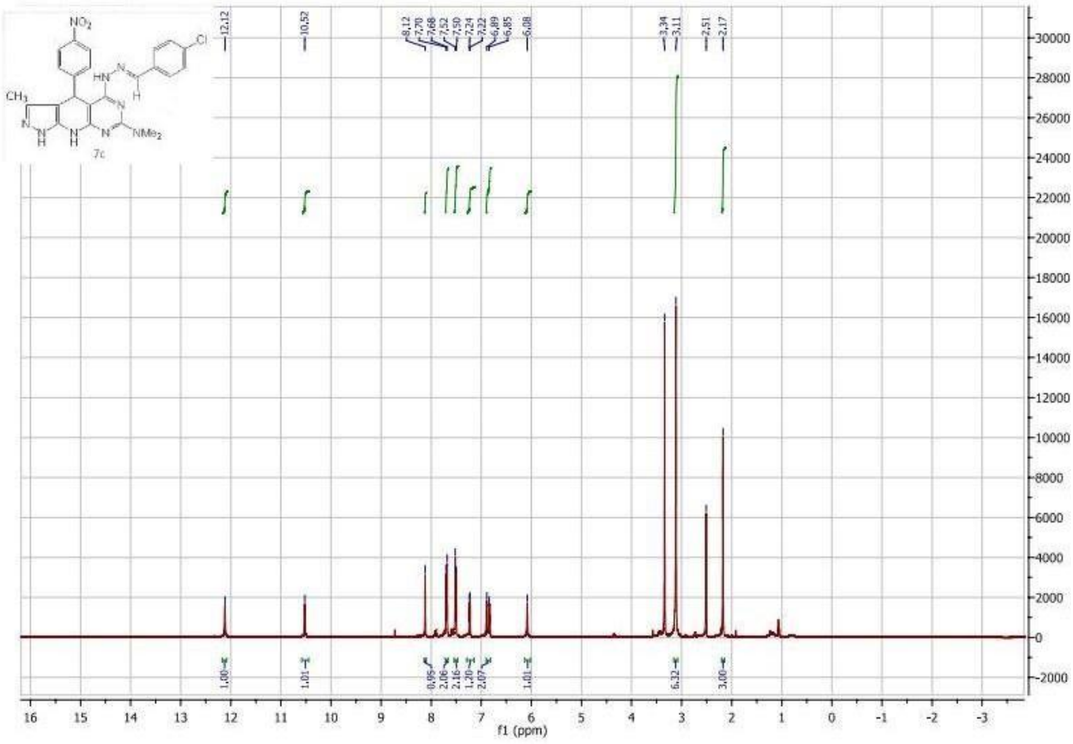

Chart S10: ^{1}H NMR of Compound **7c.**

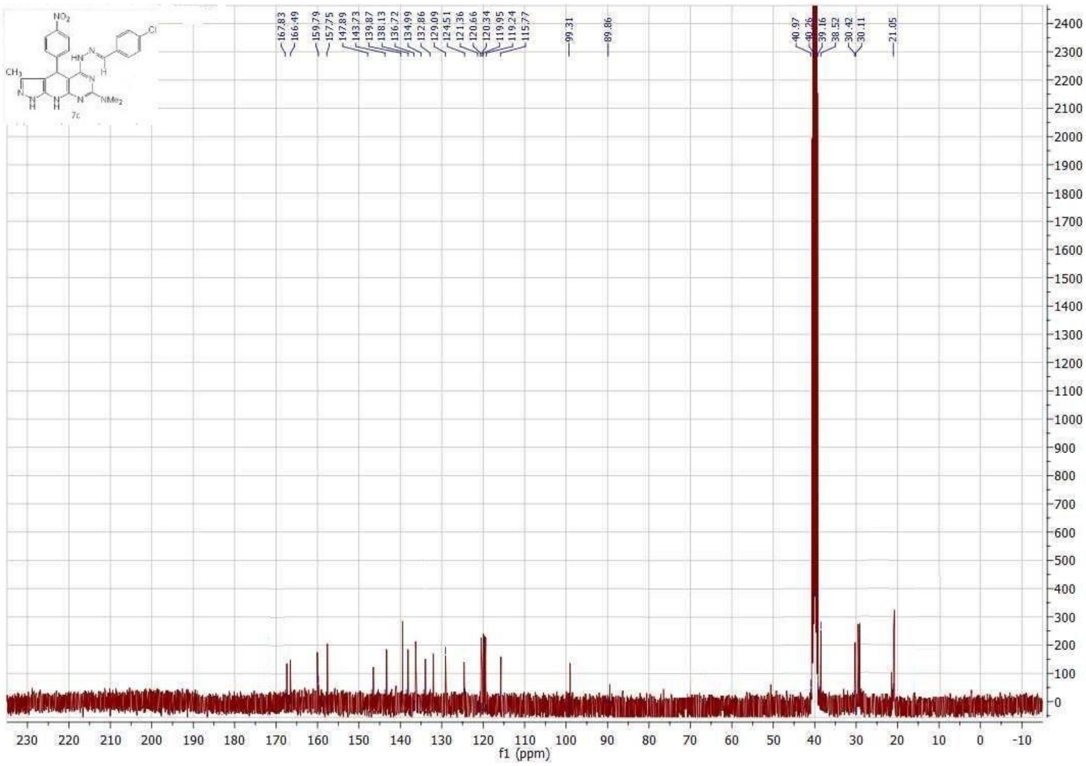

Chart S11: ^{13}C NMR of Compound **7c.**

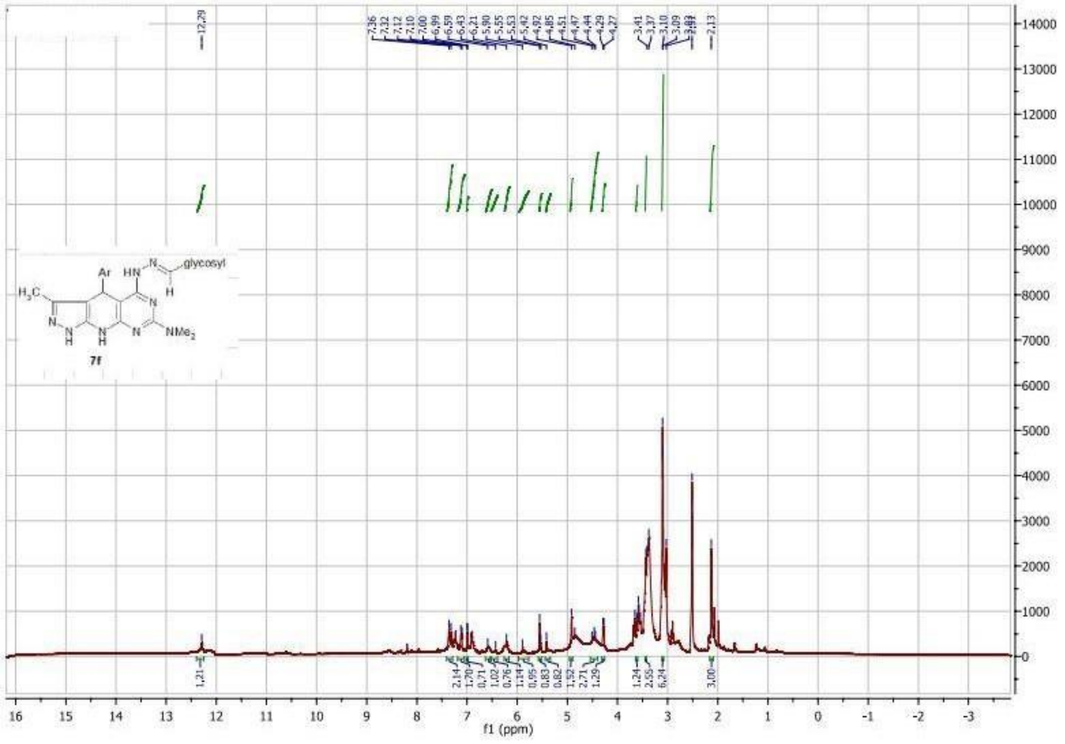

Chart S12: ^{1}H NMR of Compound **7f.**

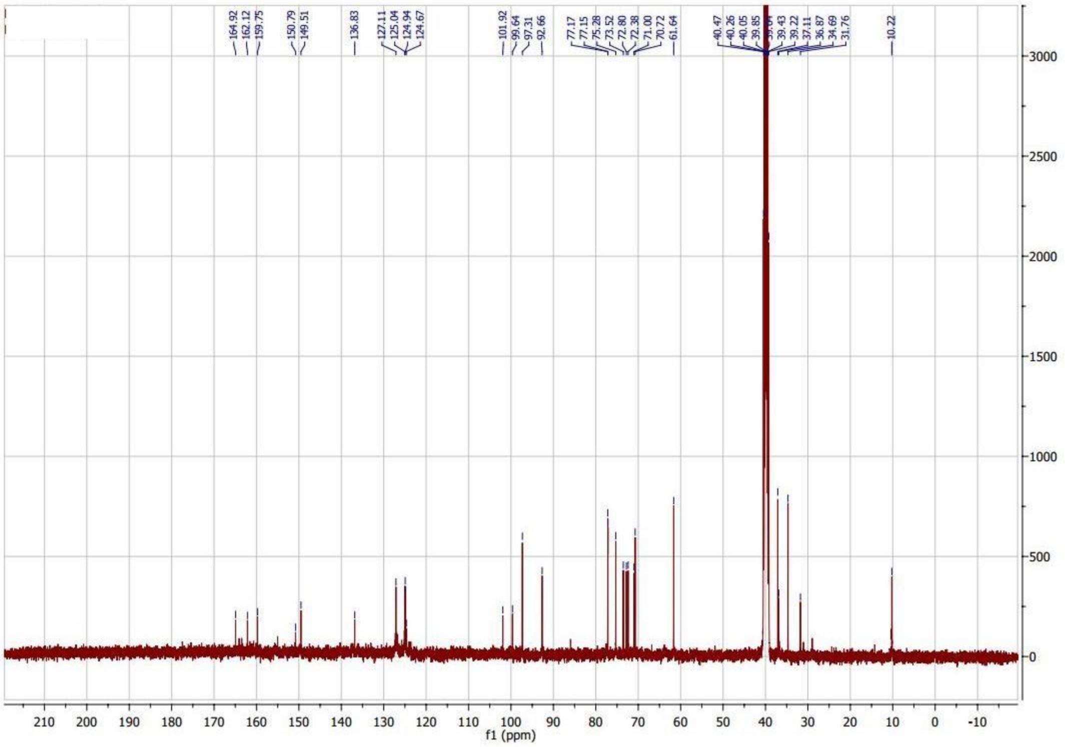

Chart S13: ^{13}C NMR of Compound **7f.**

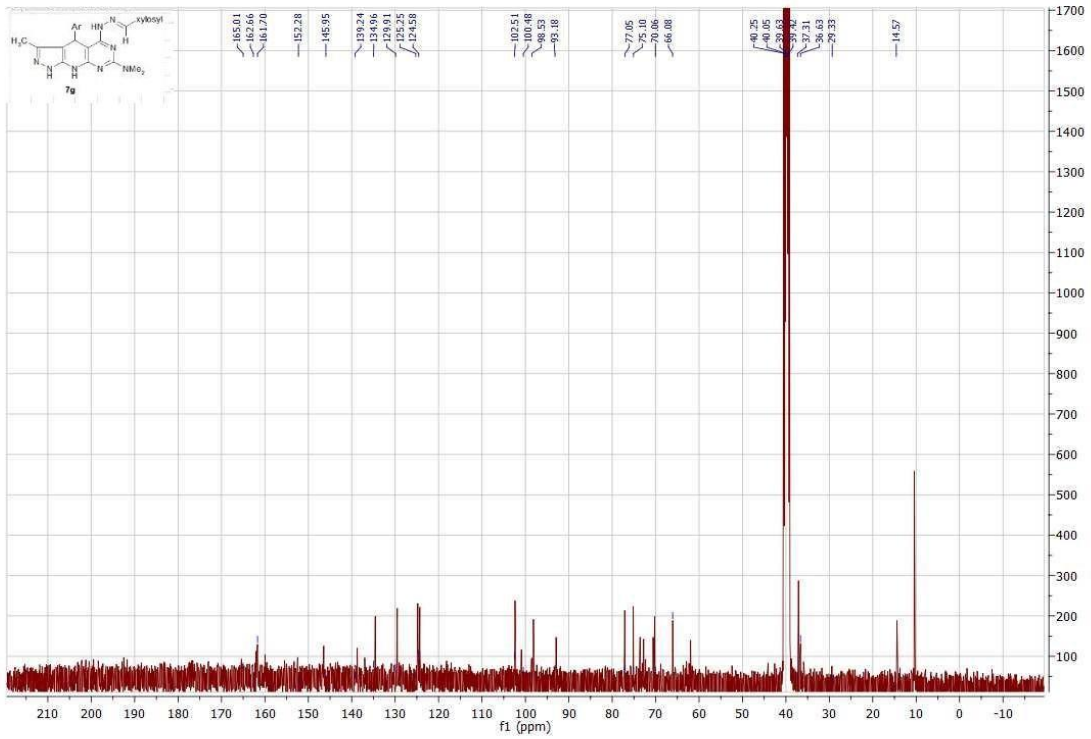

Chart S14: ^{13}C NMR of Compound **7g.**

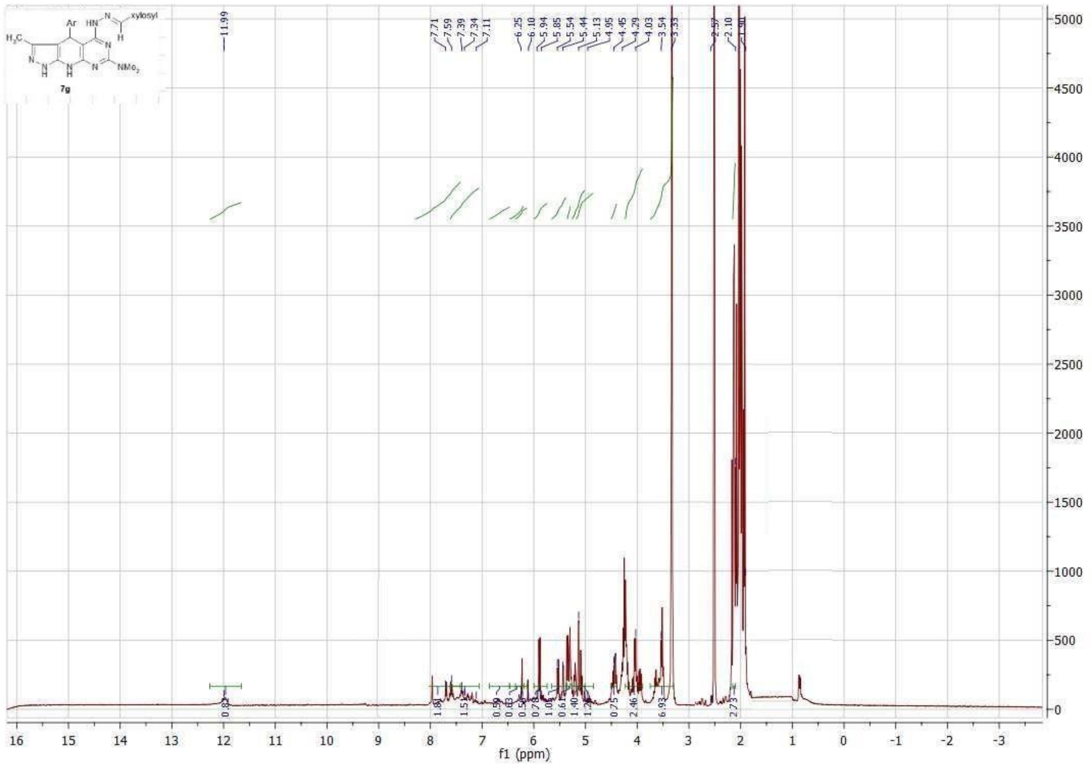

Chart S15: ^{1}H NMR of Compound **7g.**

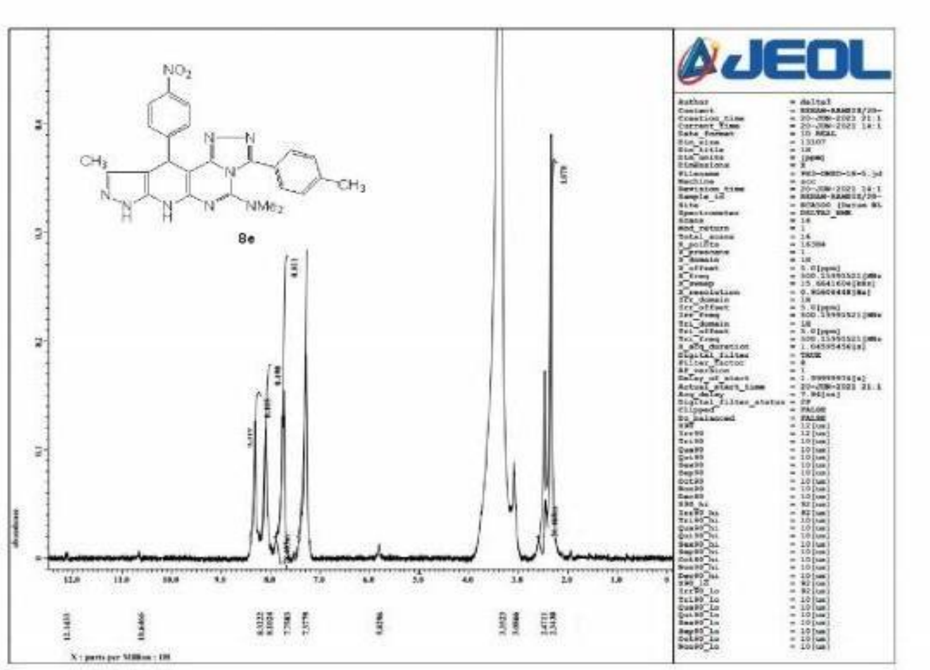

Chart S16: ^{1}H NMR of Compound **8e.**

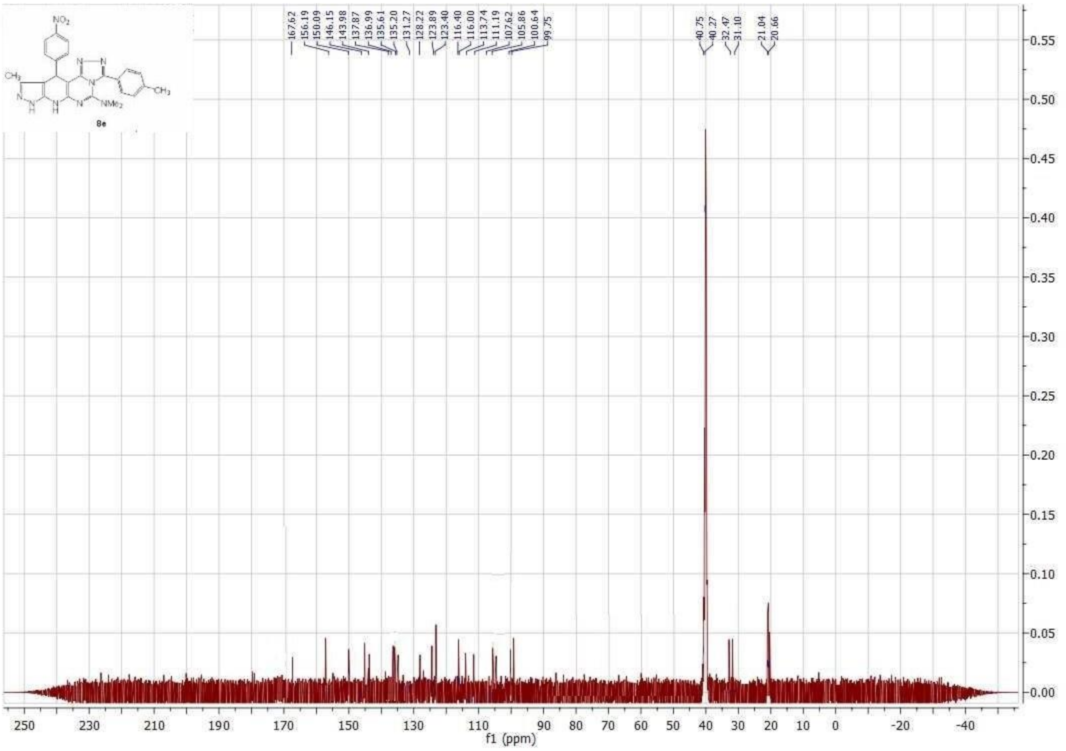

Chart S17: ^{13}C NMR of Compound **8e.**

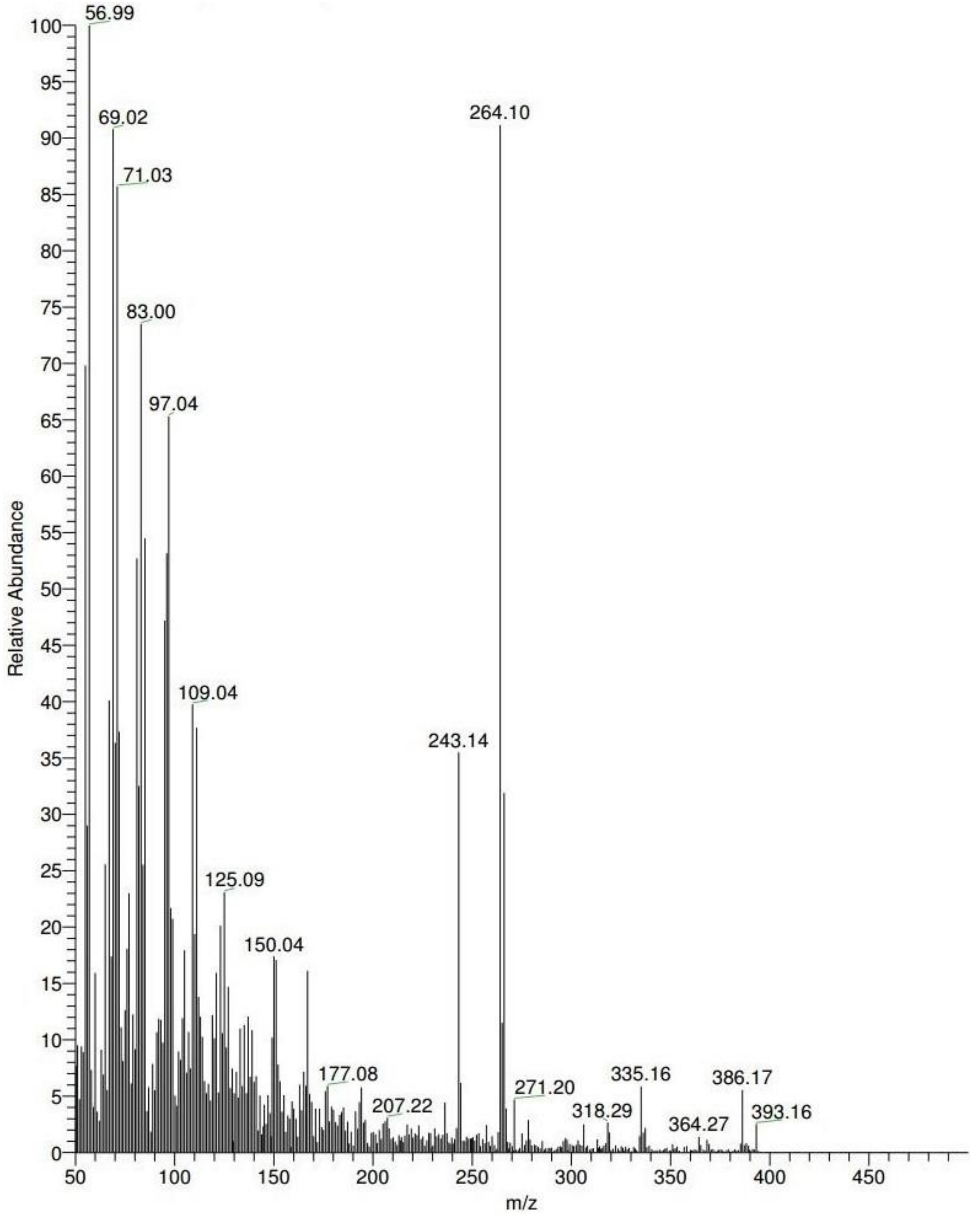

Chart S18: MS of Compound **11.**

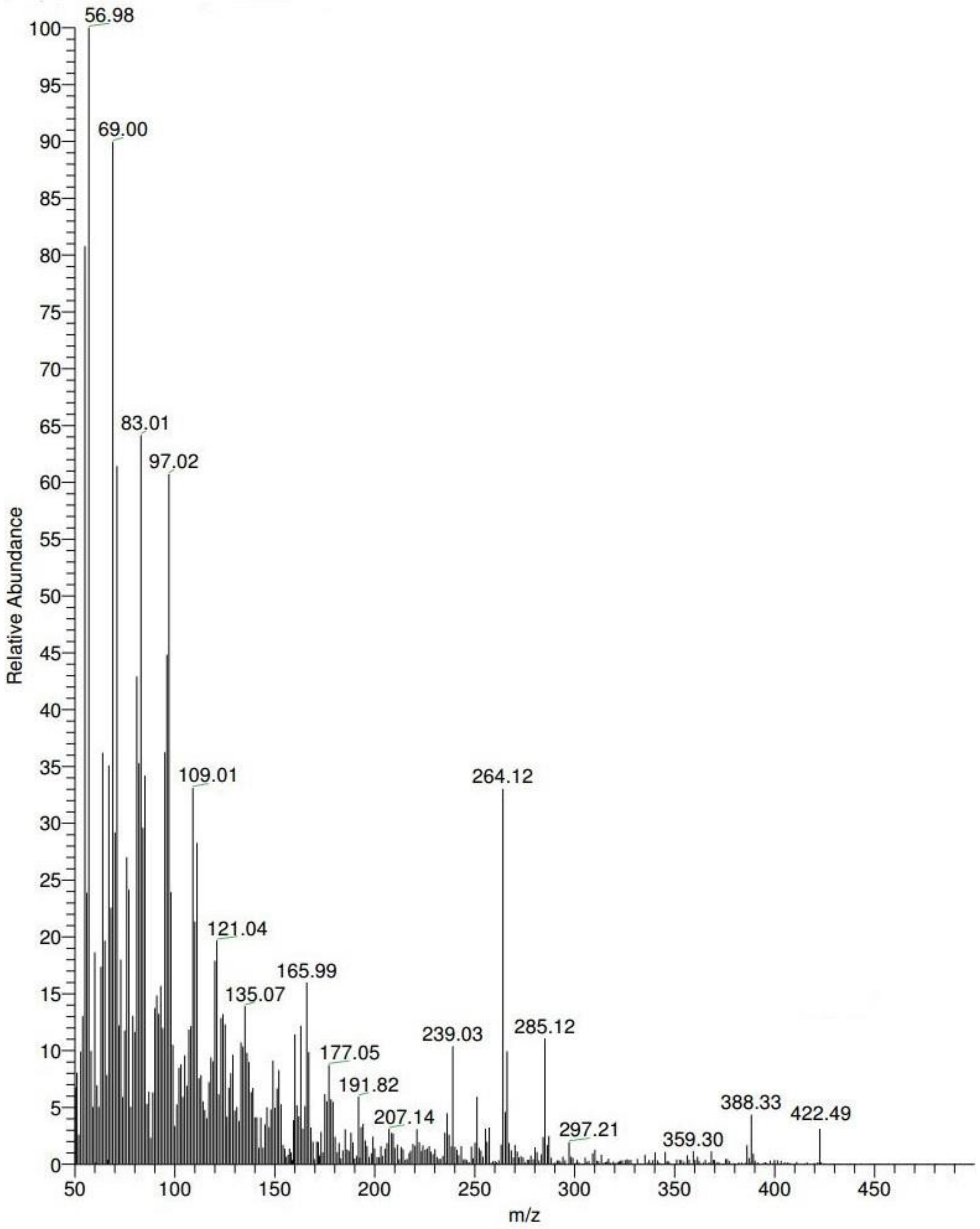

Chart S19: MS of Compound **12**

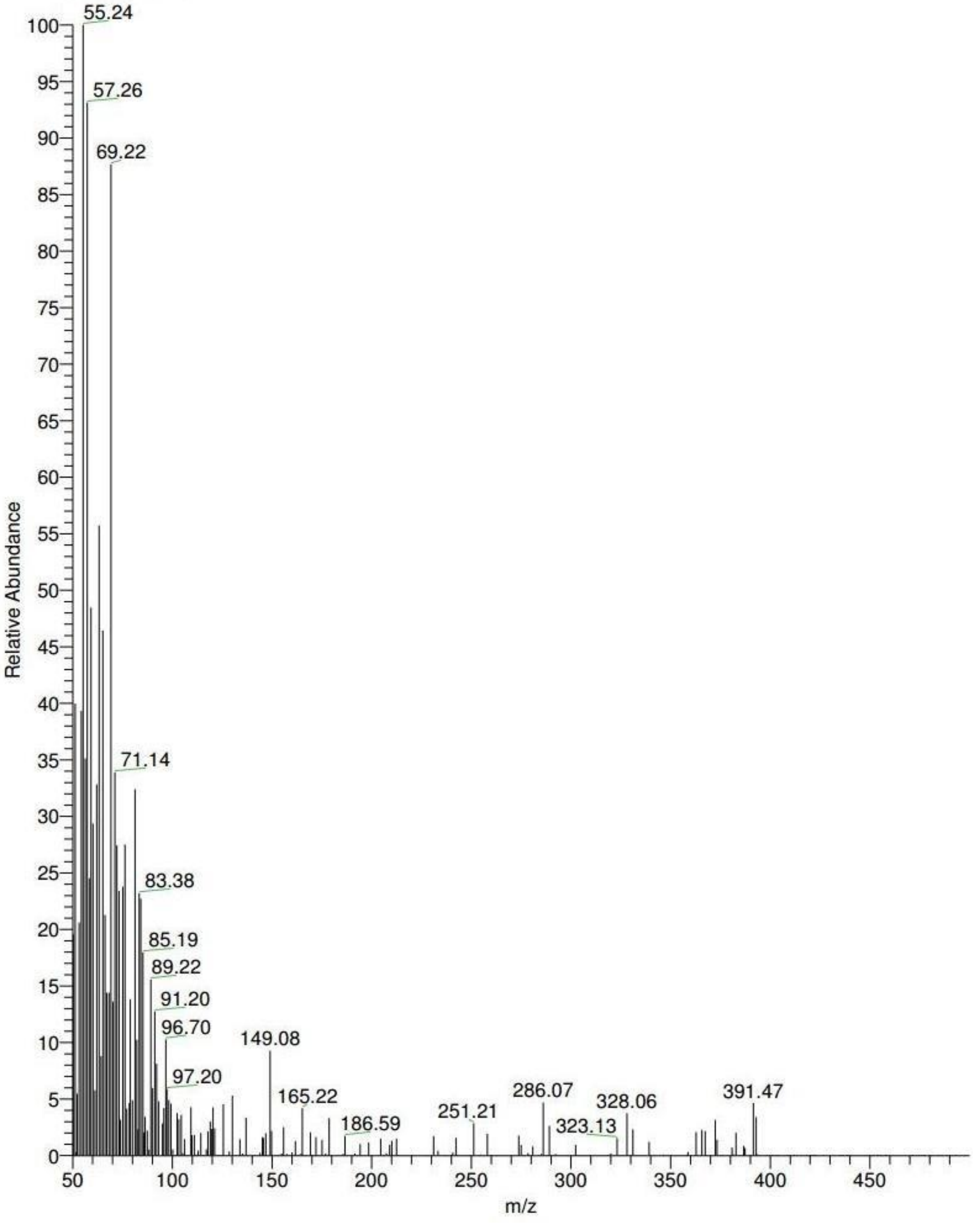

Chart S20: MS of Compound **13.**

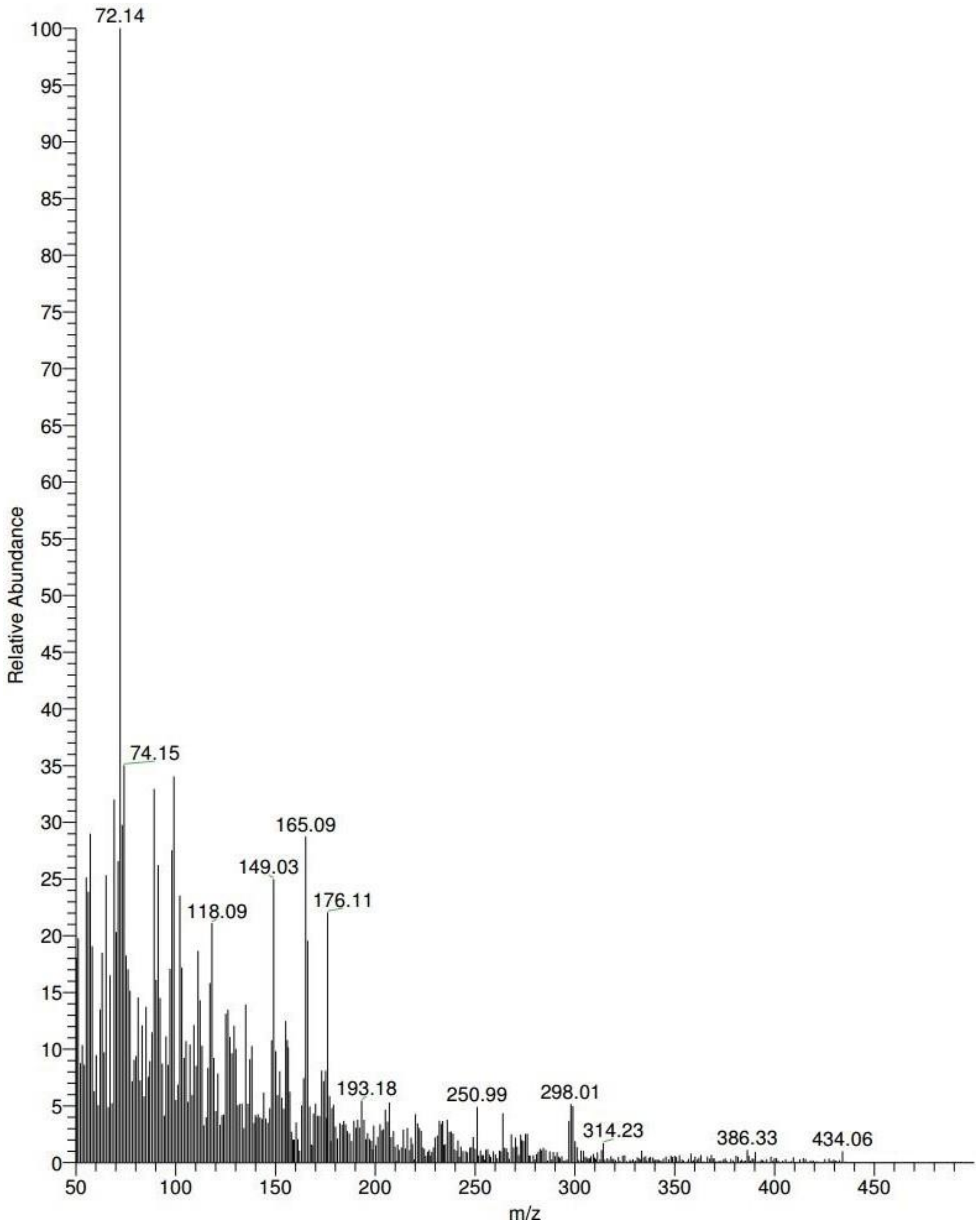

Chart S21: MS of Compound **14.**
